# Correction of Down syndrome and Edwards syndrome aneuploidies in human cell cultures

**DOI:** 10.1093/dnares/dsv016

**Published:** 2015-08-31

**Authors:** Tomokazu Amano, Emiko Jeffries, Misa Amano, Akihiro C. Ko, Hong Yu, Minoru S. H. Ko

**Affiliations:** 1 Elixirgen, LLC, Science + Technology Park at Johns Hopkins, 855 N Wolfe Street, Suite 621, Baltimore MD 21205-1511, USA; 2 Department of Systems Medicine, Keio University School of Medicine, 35 Shinanomachi, Shinjuku, Tokyo 160, Japan

**Keywords:** aneuploidy, Down syndrome, Edwards syndrome, ZSCAN4, human fibroblast cells

## Abstract

Aneuploidy, an abnormal number of chromosomes, has previously been considered irremediable. Here, we report findings that euploid cells increased among cultured aneuploid cells after exposure to the protein ZSCAN4, encoded by a mammalian-specific gene that is ordinarily expressed in preimplantation embryos and occasionally in stem cells. For footprint-free delivery of ZSCAN4 to cells, we developed ZSCAN4 synthetic mRNAs and Sendai virus vectors that encode human ZSCAN4. Applying the ZSCAN4 biologics to established cultures of mouse embryonic stem cells, most of which had become aneuploid and polyploid, dramatically increased the number of euploid cells within a few days. We then tested the biologics on non-immortalized primary human fibroblast cells derived from four individuals with Down syndrome—the most frequent autosomal trisomy of chromosome 21. Within weeks after ZSCAN4 application to the cells in culture, fluorescent *in situ* hybridization with a chromosome 21-specific probe detected the emergence of up to 24% of cells with only two rather than three copies. High-resolution G-banded chromosomes further showed up to 40% of cells with a normal karyotype. These findings were confirmed by whole-exome sequencing. Similar results were obtained for cells with the trisomy 18 of Edwards syndrome. Thus a direct, efficient correction of aneuploidy in human fibroblast cells seems possible *in vitro* using human ZSCAN4.

## 1. Introduction

Inborn deviations from the normal diploid cohort of 46 chromosomes, ‘aneuploidy’, causes severe problems in human development, growth, and function.^1–3^ Aneuploidy is caused by errors in chromosome segregation during meiosis and mitosis,^2^ and is frequently found in cancer cells.^1,4^ The most common aneuploidy sustained in live births is trisomy 21—three copies of chromosome 21 resulting in Down syndrome (DS).^5,6^ This complex developmental disorder involves congenital heart defects, haematopoietic disorders, early-onset Alzheimer's disease, cognitive impairment, and premature ageing, including telomeres shorter than those of age-matched people without DS.^6–9^

Although aneuploidy has conventionally been deemed irremediable, some researchers have recently aimed to develop ‘chromosome therapy’ for DS.^10,11^ One group has eliminated one copy of chromosome 21 by inserting a TK-NEO marker that can be selected against,^11^ and another has inactivated one copy of chromosome 21 by inserting into it a copy of the XIST gene that normally inactivates one of the two X chromosomes in female cells.^10^ These procedures have thus provided proof of principle, but have not been applied to non-immortalized primary cells due to their requirement of elaborate and cumbersome multistep genetic engineering. These limitations could be partially overcome by modifying induced pluripotent stem (iPS) cells from people with DS,^12^ but comparable culture and genetic manipulation before therapeutic intervention would still be required.^10,11^ A comparable limitation exists for a similar approach in which spontaneous correction of ring chromosomes occurs during the generation of human iPS cells from an individual with Miller–Dieker syndrome.^13^

In contrast to these complex protocols, an ideal procedure would correct aneuploidy by simply treating cells directly with small molecules or biologics that would leave no genetic alteration or footprint in cells and would thus make the intervention more tractable.

We have taken the first steps towards such a procedure based on the use of a mammalian-specific gene, Zscan4 (zinc finger and SCAN domain containing 4). We originally identified the gene as one expressed specifically at the 2-cell stage of mouse preimplantation embryos.^14^ We further showed that mouse Zscan4 is infrequently expressed in undifferentiated mouse embryonic stem (ES) cells, resulting in 1–5% of Zscan4-positive (Zscan4+) cells in culture at a given time.^14,15^ Mouse Zscan4 is also required for genome stability and maintenance of a normal karyotype in mouse ES cells.^15^ The forced expression of Zscan4 extends telomeres rapidly within a few days by a telomerase-independent recombination mechanism.^15^ Intriguingly, we found that forced transient expression of mouse Zscan4 (i.e. for a day) from a doxycycline-inducible transgene improves karyotypes and developmental potential, which otherwise characteristically deteriorate during the long-term culture of mouse ES cells.^16^ These observations suggested that Zscan4 might have potential therapeutic applications, though findings with mouse Zscan4 in mouse models might not be translatable to humans. The human equivalent, ZSCAN4, was also known to have a unique expression pattern,^17,18^ but had not been further characterized. In particular, it was not known whether it could function in differentiated somatic cells that are the putative target of therapies. For example, somatic cells might lack partner proteins that are present in ES cells and preimplantation embryos. Also, all the previous work had used a mouse Zscan4-transgene integrated into the genome, a scenario very different from the goal of accomplishing human ZSCAN4 function without requiring complicated genetic engineering of cells.

Here, we report the development of novel biologics—synthetic mRNAs and Sendai virus vectors encoding human ZSCAN4—which can efficiently increase cells with normal karyotypes in mouse ES cells as well as in non-immortalized primary human fibroblast cells derived from people with DS or Edwards syndrome (Trisomy 18).^19^ These results motivate further work to test possible applications of ZSCAN4 biologics to treat chromosome abnormalities.

## 2. Materials and methods

### 2.1. Development of ZSCAN4 biologics

From the perspective of developing therapeutics, it is desirable to overexpress ZSCAN4 without stably integrating an exogenous transgene DNA into the cells, which could cause insertion-based mutations. Because Zscan4 is only required transiently, we decided to test the direct delivery of mRNAs into the cells, which are translated into proteins. We chose two mRNA delivery methods: Synthetic mRNAs (Syn-mRNAs) and Sendai virus (SeV) vectors.

For the synthesis of modified mRNA, mRNA synthesis was performed as reported previously.^20^ In brief, mRNAs were synthesized by *in vitro* transcription of template DNAs encoding mouse Zscan4c, human ZSCAN4, or Green Fluorescent Protein (GFP) with modified mixtures of dNTP to increase RNA stability as well as translation efficiency in mammalian cells.

For SeV vectors, we used a non-transmissible vector that lacks the F protein.^21^ Although wild-type SeV is known for its function of causing cell fusions, the SeV vectors used here lack the capacity for cell fusion.^21^ Temperature-sensitive variants of non-transmissible SeV vectors (SeV18/ΔF-TS7 and SeV18/ΔF-TS15),^22^ which express mouse Zscan4c, human ZSCAN4, or a green fluorescent protein variant—Azami-Green (AG, a control), were custom-made (DNAVEC Corporation, Tsukuba, Japan). It has been shown that SeV-TS7 is functional at 35°C and weakly functional at 37°C, but not at the non-permissive temperature of 38°C or 39°C, whereas SeV-TS15 is functional at 35°C, but not at 37°C, though their temperature-profiles are slightly different.^22^

### 2.2. Mouse ES cells

We used a previously established mouse ES cell line, MC1ZE16, which was cultured in the standard condition.^16^ In brief, the cells were grown at 37°C in 5% CO_2_ in complete ES medium: DMEM (Invitrogen), 15% heat inactivated fetal bovine serum (FBS) (Life Technologies), 1000 U ml^−1^ leukaemia inhibitory factor (ESGRO), 1 mM sodium pyruvate, 0.1 mM non-essential amino acids, 2 mM GlutaMAX, 0.1 mM β-mercaptoethanol, and penicillin/streptomycin (50 U/50 mg ml^−1^). The medium was changed daily.

### 2.3. Primary fibroblast cells derived from people with DS and Edwards syndrome

Fibroblast cells isolated from four individuals with DS (Trisomy 21) were purchased from the Coriell Cell Repository (NJ, USA). Their catalogue numbers were AG05397 (47,XY,+21), AG08942 (47,XY,+21), AG05024 (47,XX,+21), and AG06872 (47,XX,+21). Fibroblast cells isolated from an individual with Edwards syndrome (Trisomy 18) were also purchased from the Coriell Cell Repository (NJ, USA). Their catalogue number was AG12614 (47,XX,+18). All of these fibroblast cells were non-immortalized primary fibroblast cells. All of these anonymized cells were used according to the guidelines provided by the Coriell Cell Repository. These cells were cultured in the standard culture condition as instructed by the Coriell Cell Repository.

### 2.4. Transfection of mouse ES cells with Syn-mRNAs

Five to 6 h before transfection, 2 × 10^5^ cells per well were plated in a 6-well plate. Transfection was performed using 1 µg of Syn-mRNA and 5 µl of RNAiMax (Invitrogen) according to the manufacturer's instructions.

### 2.5. Infection of mouse ES cells with SeV vectors

Mouse ES cells were plated on a gelatin-coated 6-well plate and then infected with either SeV-mZscan4-TS15 or SeV-hZSCAN4-TS15 at a multiplicity of infection (MOI) of 25. Then they were cultured at 35°C for 3 days, and then transferred to 37°C. Cells were passaged on days 2 and 4. The MOI of 25 was determined after optimizing the SeV doses on mouse ES cells (Supplementary Fig. S1).

### 2.6. Transfection of human fibroblast cells with Syn-mRNAs

Human non-immortalized primary fibroblast cells (5 × 10^4^cells/well) were plated in a 6-well plate and then transfected with 1 µg of Syn-mRNAs (Syn-hZSCAN4 or Syn-GFP) using 5 µl of Lipofectamine (RNAiMax: Life Technologies, CA, USA). In addition to cells transfected with a Syn-GFP, non-transfected cells were also used as a control. The dose of Syn-mRNAs (i.e. 1 µg for 5 × 10^4^cells/well in a 6-well plate) was determined after optimization (Supplementary Fig. S2).

### 2.7. Infection of human fibroblast cells with SeV vectors

Fibroblast cells were plated in a 6-well plate (5 × 10^4^cells/well) and immediately treated with the SeV vectors described above at an MOI of 25 (day 0). Medium was changed next day to remove the remaining SeV vectors (day 1). Cells were kept at 35°C for 7 days then transferred to 37°C (day 7). On day 7, the production of protein was monitored either by fluorescence microscopy for SeV-AG or by immunostaining for SeV-hZSCAN4. Infected cells were subcultured continuously. In the third week of treatment (on day 21, 23, or 24), the number of chromosome 21 was counted by fluorescent *in situ* hybridization (FISH). The MOI of 25 was determined after optimizing the SeV doses on human fibroblast cells (Supplementary Fig. S3).

### 2.8. Chromosome FISH

We used a chromosome 21-specific probe (CHR21-10-GR) and a chromosome 18-specific probe (CHR18-10-GR) purchased from Empire Genomics (Buffalo, NY, USA). CHR21-10-GR hybridized to the centromeric region long-arm band 21q21.1 and CHR18-10-GR hybridized to the short-arm band 18p11. Chromosome FISH was carried out according to the manufacture's protocol. In brief, cells were attached to a glass slide, which was treated with pre-warmed denaturation buffer at 73°C for 5 min and placed in 70, 85, and 100% ethanol at room temperature for 1 min serially. Before hybridization, probes were denatured at 73°C for 5 min and placed at 37°C for 15 min, then applied to the slides, which were then incubated at 37°C for 16 h for hybridization. After washing, slides were visualized under a microscope using the appropriate fluorescent filter set.

### 2.9. Karyotype analyses of mouse ES cells

Mouse ES cells were treated with 0.1 μg/ml Colcemid (Gibco) for 3 h to enrich metaphase cells, and then the cells were exposed to 0.56% KCl for 10 min and fixed by 3:1 methanol:glacial acetic acid. Slides were stained with Giemsa stain before observations.

### 2.10. Karyotype analyses of human cells

High-resolution G-banded karyotyping was carried out in a blinded manner by Cell Line Genetics, Inc. (Madison, WI, USA). Live cell cultures were sent to Cell Line Genetics, Inc. and the chromosome analyses of 40 cells per sample were requested. Comprehensive interpretation of results was provided by their scientist and the reports were signed by a clinically certified cytogeneticist. The service complies with FDA Good Laboratory Practice (GLP).

### 2.11. Measurement of telomere length

Genomic DNAs were extracted by using the DNeasy Kit (Qiagen) and quantified by Nanodrop. Telomere length was measured by a quantitative real-time PCR method described previously.^23^ In brief, qRT–PCR was performed on the StepOnePlus Real-Time PCR System (Life Technologies) by using SYBR Green PCR Master Mix (Life Technologies). For telomere PCR, 50 nM Tel1 (5′-GGTTTTTGAGGGTGAGGGTGAGGGTGAGGGTGAGGGT-3′) and 300 nM Tel2 (5′-TCCCGACTATCCCTATCCCTATCCCTATCCCTATCCCTA-3′) were used. For a single copy gene (36B4) PCR, 300 nM 36B4u (5′-CAGCAAGTGGGAAGGTGTAATCC-3′) and 500 nM 36B4d (5′-CCCATTCTATCATCAACGGGTACAA-3′) were used. For both PCR amplifications, the conditions were 95°C incubation for 10 min followed by 35 cycles of 95°C 15 s, and 58°C 1 min. Standard curves were produced by the serial dilution of reference DNAs from 100 to 3.125 ng. A relative T/S ratio (Telomere/Single copy gene) was generated to indicate the relative telomere length for each sample.

### 2.12. Quantitative RT–PCR

Total RNA was extracted using the RNeasy Mini kit (QIAGEN). For cDNA synthesis, 2.5 µg of total RNAs was reverse transcribed using random 6-mer primers by PrimeScript 1st strand cDNA Synthesis Kit (Takara) according to the manufacturer's instructions. qPCR analysis was performed using 50 ng of cDNAs per well in triplicate with SYBR green PCR master mix (Life Technologies) according to the manufacturer's instructions. Primers used: hZSCAN4 5′-UTR (forward, 5′-ACCAAGGAGCTTGCAGTTT-3′; reverse, 5′-TTCAGTCTCTTGCCTTGTGTC-3′); Tmem92 (forward, 5′-CCTGCTTTCCAGTCTCCAAG-3′; reverse, 5′-TGACCAAGAACCTAAAGCGG-3′). Reactions were performed on a StepOnePlus Real-Time PCR System (Life Technologies). Fold induction was calculated by the ΔΔCt method.

### 2.13. Immunohistochemistry

Cells were plated on sterilized cover slides placed in 12-well plates. The cover slides were taken out from the plate, washed with PBS, and fixed with 4% paraformaldehyde for 10 min at room temperature. After blocking in 10% normal goat serum and 0.2% Triton-X for 30 min, cells were incubated with mouse anti-human ZSCAN4 antibodies (Sigma: catalogue number: SAB1400842) at dilution of 1:1000 for 30 min and then incubated with Alexa 555 goat anti-mouse IgG antibodies (Invitrogen) for 30 min. Nuclei were visualized with DAPI staining.

### 2.14. Whole-exome sequencing analyses

Genome-wide exome sequencing was carried out in a blinded manner by a commercial sequencing service (Empire Genomics, LLC). In brief, genomic DNA was extracted from cell pellets using the Gentra PureGene Blood Core Kit B. Whole-exome sequencing was performed on genomic DNA using the AgilentSureSelectXT Human All Exon V4 kit. Paired-end 100 bp read lengths were sequenced on an Illumina Hiseq 2000 next-generation sequencer to achieve 50-fold coverage per sample. Resultant FastQ files were processed in the standard manner (http://seqanswers.com/wiki/How-to/exome_analysis). In brief, sequences were aligned to the human genome (hg19) and generated SAI files were converted to SAM files by the BWA tool.^24^ The SAM files were converted to BAM files and sorted by Picard (http://picard.sourceforge.net). PCR duplicates were marked by Picard. Local realignment around indels was carried out by the Genome Analysis Tool Kit (GATK).^25^ The mate information for the paired end data was fixed by Picard. Quality score recalibration was carried out by the GATK. Raw SNP calls were generated and filtered by the GATK. The resultant VCF files were viewed and analysed by VarSifter.^26^ Also, VCF files and BAM files were viewed and analysed by the IGV.^27^

The following are indicators of quality and quantity of sequences generated from three samples—non-treated control cells, SeV-AG-treated control cells, and SeV-hZSCAN4-treated cells. These alignment summary reports were generated by a ‘flagstat’ command of SAMtools^28^ (Version 0.1.19-44428cd).

Non-treated control cells:

51 862 788 + 0 in total (QC-passed reads + QC-failed reads)
0 + 0 duplicates
51 473 127 + 0 mapped (99.25%:-nan%)
51 862 788 + 0 paired in sequencing
25 931 394 + 0 read1 --> Number of tags in paired end 1
25 931 394 + 0 read2 --> Number of tags in paired end 2
50 909 452 + 0 properly paired (98.16%:-nan%)
51 384 595 + 0 with itself and mate mapped
88 532 + 0 singletons (0.17%:-nan%)
267 151 + 0 with mate mapped to a different chr
256 545 + 0 with mate mapped to a different chr (mapQ ≥ 5)

SeV-AG-treated control cells:

66 090 080 + 0 in total (QC-passed reads + QC-failed reads)
0 + 0 duplicates
64 741 280 + 0 mapped (97.96%:-nan%)
66 090 080 + 0 paired in sequencing
33 045 040 + 0 read1 --> Number of tags in paired end 1
33 045 040 + 0 read2 --> Number of tags in paired end 2
64 116 010 + 0 properly paired (97.01%:-nan%)
64 615 277 + 0 with itself and mate mapped
126 003 + 0 singletons (0.19%:-nan%)
288 571 + 0 with mate mapped to a different chr
276 632 + 0 with mate mapped to a different chr (mapQ ≥ 5)

SeV-hZSCAN4-treated cells:

49 978 930 + 0 in total (QC-passed reads + QC-failed reads)
0 + 0 duplicates
49 615 372 + 0 mapped (99.27%:-nan%)
49 978 930 + 0 paired in sequencing
24 989 465 + 0 read1 --> Number of tags in paired end 1
24 989 465 + 0 read2 --> Number of tags in paired end 2
49 113 676 + 0 properly paired (98.27%:-nan%)
49 532 552 + 0 with itself and mate mapped
82 820 + 0 singletons (0.17%:-nan%)
242 624 + 0 with mate mapped to a different chr
233 078 + 0 with mate mapped to a different chr (mapQ ≥ 5)

To detect the changes of chromosome numbers in the cells, we used ‘AlleleBalance’—variant annotations provided by the GATK tool.^25^ AlleleBalance (AB) was calculated based on the read depth of a reference allele (REF) and an alternative allele (ALT): homozygous ‘ABHom’ was obtained by dividing the number of ALT alleles by the total number of alleles; heterozygous ‘ABHet’ was obtained by dividing the number of REF alleles by the total number of alleles. In theory, the value of the ABHom should be close to 1.00 for homozygous alleles and the value of the ABHet should be close to 0.5 for heterozygous alleles.

To analyse the AlleleBalance, all of the information from the VCF files was extracted into Microsoft Excel format. Because it is known that sequence coverage will not be uniform on all the exons, we only analysed the common variant alleles that were detected in three samples. We also filtered out low-quality sequences and variants with low sequence coverage. Because the GATK does not provide ABHom and ABHet calculation for INDELs, we also filtered out INDELs. We then removed homozygous alleles (ABHom >0.5: for almost all of them, ABHom >0.95). ABHet scores of the remaining alleles were used to generate histograms for all of the chromosomes separately.

According to the guidelines of the provider of cells (The Coriell Cell Repository), sequences obtained from donor-derived cells cannot be deposited into the public database to protect the donors’ privacy. Therefore, we will provide the sequence data to researchers upon request on the condition that the data will be handled according to the same strict guidelines.

## 3. Results

### 3.1. ZSCAN4 biologics improve karyotype in mouse ES cells

In order to create an integration-free and footprint-free method to express a Zscan4 gene ectopically, we developed synthetic mRNAs (Syn-mRNAs). Syn-mRNAs are not converted to DNAs, and thus cannot integrate into the host genome.^20,29^ The production of protein from Syn-mRNAs peaks ∼12 h after transfection and then declines precipitously^20^ (see Materials and methods for further details). We treated mouse ES cells once with either Syn-hZSCAN4 or Syn-GFP (control), and harvested the cells 4 days later for karyotype analyses (Fig. 1A). Representative images of abnormal and normal karyotypes are shown in Fig. 1B. This mouse ES cell line was used because of its relatively low percentage of euploid cells after long-term cell culture.^16^ Non-treated control cells showed only ∼18% euploidy (Fig. 1C).^16^ Strikingly, the one-shot treatment of the cells with Syn-hZSCAN4 rapidly increased the percentage of euploid cells to 29%, whereas the treatment of cells with a control Syn-GFP did not change the percentage of euploidy (Fig. 1C). qRT–PCR analyses showed that marker genes known to be up-regulated by the overexpression of Zscan4^15,16^ were also up-regulated in the Syn-hZSCAN4-treated cells (Fig. 1D). This confirmed that Zscan4 products indeed functioned in a manner similar to a Zscan4-transgene studied in a previous work.^16^

**Figure 1.**
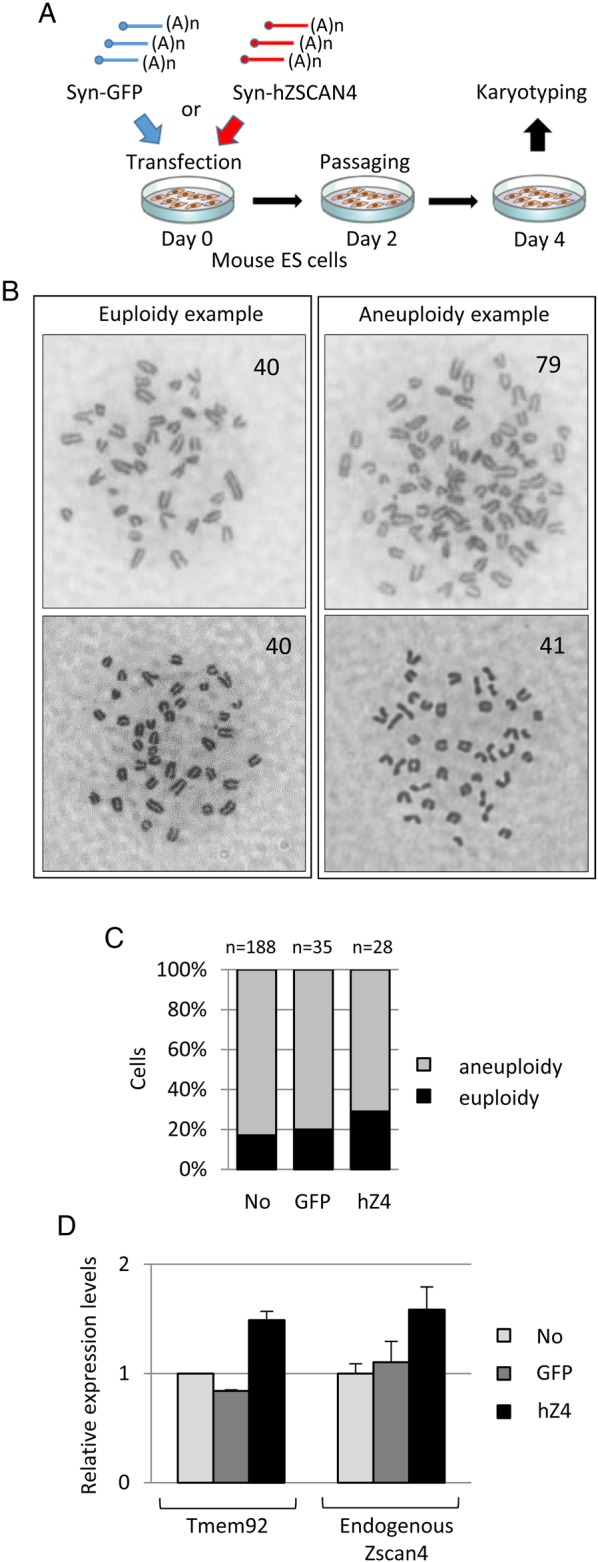
Analyses of mouse ES cells treated with synthetic mRNAs encoding human ZSCAN4. (A) Schematic overview of an experimental approach using Syn-mRNAs. (B) Examples of karyotypes: euploidy (left); aneuploidy (right). (C) The percentages of euploid cells (black box) and aneuploid cells (grey), respectively. Total numbers of examined cells were shown above each bar (*n*). Non-treated control cells (No), cells treated with Syn-GFP (GFP), and cells treated with Syn-hZSCAN4 (hZ4). (D) Quantitative RT–PCR analyses demonstrating the expression levels of Tmem92 and endogenous Zscan4 in non-treated control cells (No), cells treated with Syn-GFP (GFP), and cells treated with Syn-hZSCAN4 (hZ4). This figure is available in black and white in print and in colour at *DNA Research* online.

As an independent method for footprint-free delivery of Zscan4 products, we used a Sendai virus (SeV) vector. SeV vectors are based on a single-stranded RNA virus of the Paramyxovirus subfamily that completes its infection cycle as RNA (i.e. without involvement of DNAs). Therefore, a recombinant gene of interest transduced by SeV vector will not be integrated into the host genome. SeV vectors have been judged safe for human treatment, and several ongoing clinical trials utilize a SeV vector to deliver RNAs to cells.^30–32^ More specifically, we used non-transmissible SeV vectors (SeV18/ΔF)^21^ and their temperature-sensitive variants (SeV18/ΔF-TS15),^22^ which express mouse Zscan4 (SeV-mZscan4-TS15), human ZSCAN4 (SeV-hZSCAN4-TS15), or the Azami-Green green fluorescent protein variant (SeV-AG-TS15, a control) (see details in Materials and methods).

We applied SeV-TS15 to mouse ES cells, incubated them at the permissive temperature of 35°C for 3 days and then at the non-permissive temperature of 37°C for 3 days before karyotype analyses (Fig. 2A). Both SeV-mZscan4-TS15 and SeV-hZSCAN4-TS15 dramatically increased the percentage of euploidy to 50%, whereas SeV-AG-TS15 did not increase the percentage of euploidy above the level of the control mouse ES cells (Fig. 2B and C). Similar to the Syn-mRNA-treatment, qRT–PCR analyses confirmed that the ZSCAN4 products delivered by the SeV-mZscan4-TS15 and SeV-hZSCAN4-TS15 functioned in mouse ES cells (Fig. 2D).

**Figure 2.**
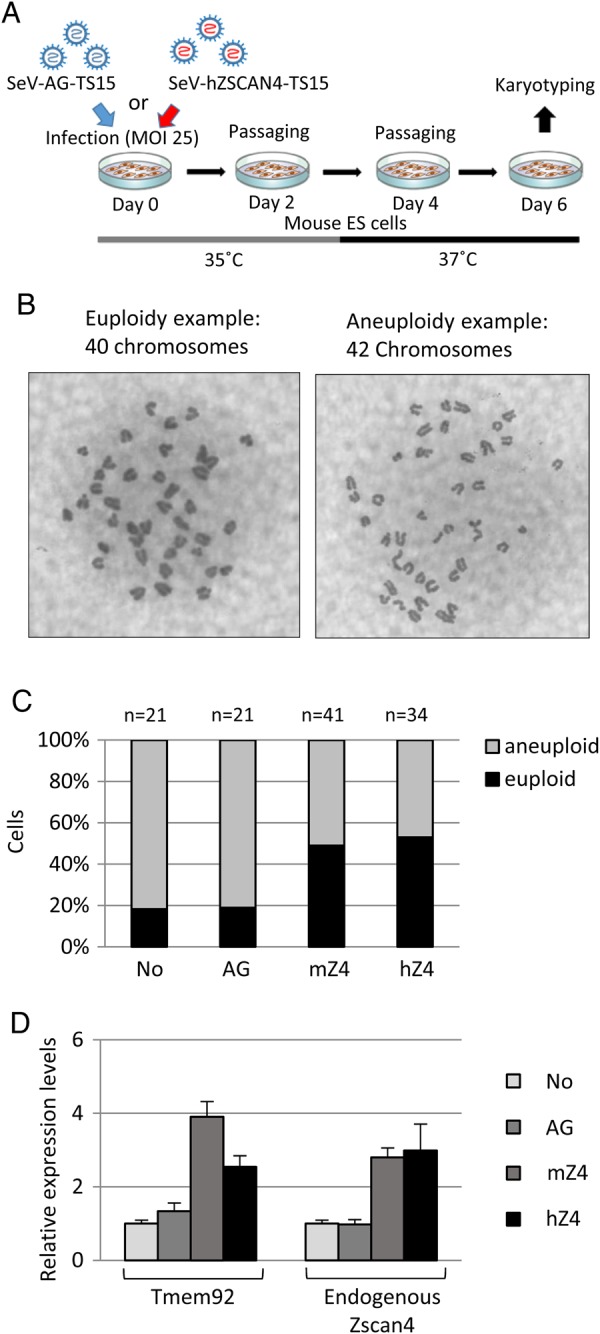
Analyses of mouse ES cells treated with Sendai virus vector encoding mouse or human ZSCAN4. (A) Schematic overview of an experimental approach using SeV vectors. (B) Examples of karyotypes: euploidy; aneuploidy. (C) The percentages of euploid cells (black box) and aneuploid cells (grey), respectively. Total numbers of examined cells were shown above each bar (*n*). Non-treated control MC1ZE cells (No), cells treated with SeV-AG-TS15 (AG), cells treated with SeV-mZscan4-TS15 (mZ4), and cells treated with SeV-hZSCAN4-TS15 (hZ4). (D) Quantitative RT–PCR analyses demonstrating the expression levels of Tmem92 and endogenous Zscan4 in non-treated control MC1ZE cells (No), cells treated with SeV-AG-TS15 (AG), cells treated with SeV-mZscan4-TS15 (mZ4), and cells treated with SeV-hZSCAN4-TS15 (hZ4). This figure is available in black and white in print and in colour at *DNA Research* online.

The rapid improvement of karyotypes in mouse ES cells using two independent ZSCAN4 delivery methods suggests that the ZSCAN4 protein itself causes the dramatic increase in euploid cells directly and efficiently. The data also indicate that not only mouse Zscan4, but also human ZSCAN4 functions in the same manner in mouse ES cells.

### 3.2. Synthetic mRNAs encoding human ZSCAN4 increase euploid cells in DS fibroblast cells

To investigate whether human ZSCAN4 biologics could correct aneuploidy in human cells, we examined the effects of synthetic mRNAs (Syn-hZSCAN4 and Syn-GFP as a control) on non-immortalized primary fibroblast cells isolated from an individual with DS (AG05397: 47,XY,+21). Cells (5 × 10^4^cells/well) were plated in a 6-well plate and then treated with 1 µg of synthetic mRNAs (Syn-hZSCAN4 or Syn-GFP) using 5 µl of Lipofectamine (see Materials and methods) (Fig. 3A). The efficiency of synthetic mRNA delivery and subsequent protein production were monitored 1 day after the treatment by fluorescence microscopy for GFP and by immunohistochemistry for ZSCAN4. We found that efficiency varied from experiment to experiment, but was within the range of 50–80% (Fig. 3B).

**Figure 3.**
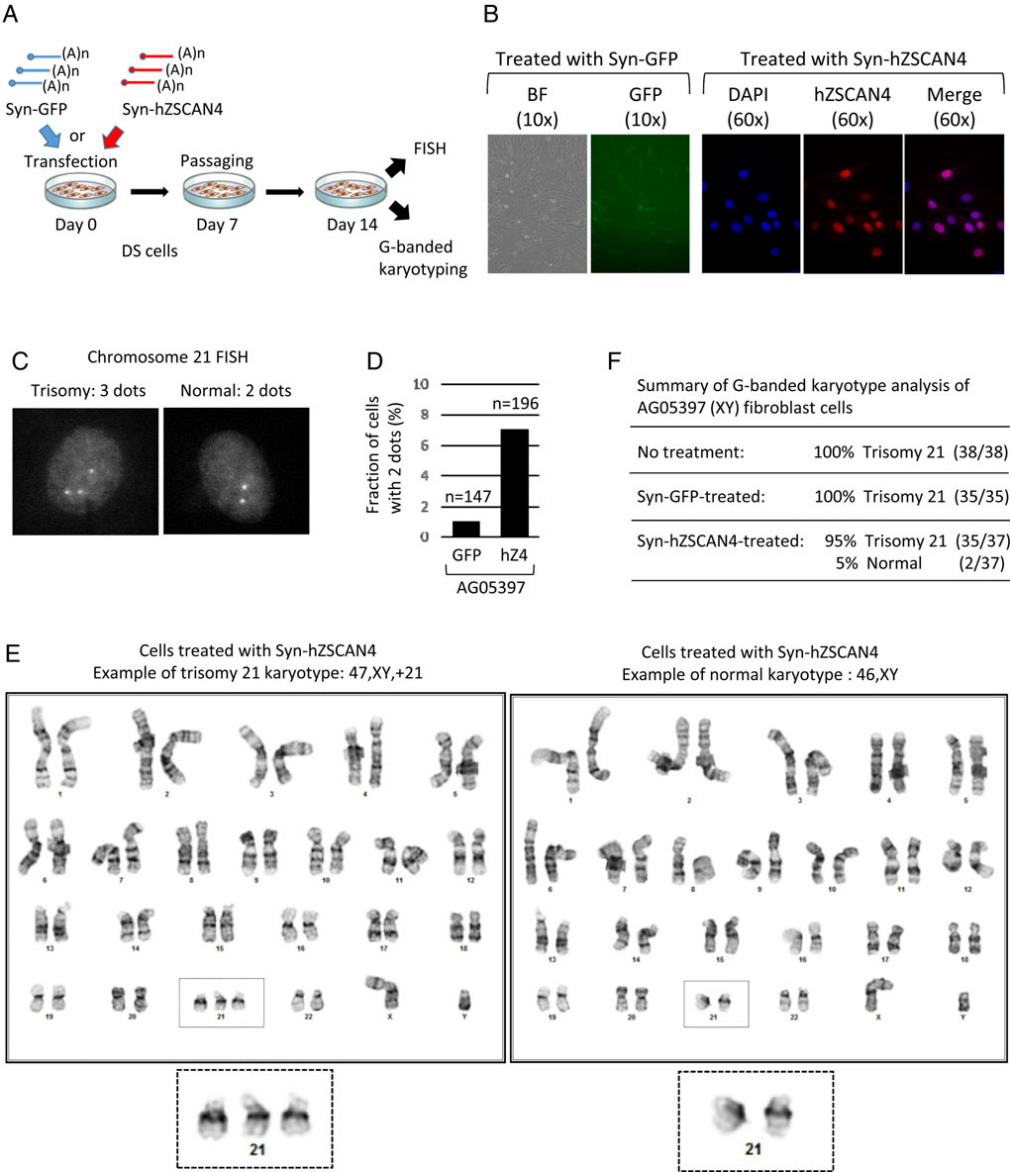
Analyses of human trisomy 21 primary fibroblast cells treated with Syn-mRNAs. (A) Schematic overview of an experimental approach using Syn-mRNAs. (B) Assessment of GFP expression by fluorescence microscopy and hZSCAN4 expression by immunohistochemistry using an antibody against human ZSCAN4 (1 day after transfection). (C) Examples of FISH using a chromosome 21-specific probe: left, three dots (three copies of chromosome 21); right, two dots (two copies of chromosome 21). (D) The fraction (%) of AG05397 cells with two dots. GFP: cells treated with Syn-GFP (*n* = 147). hZ4: cells treated with Syn-hZSCAN4 (*n* = 196). (E) Examples of high-resolution G-banded karyotype after treating AG05397 (trisomy 21) cells with Syn-hZSCAN4. Left, trisomic karyotype. Right, normal karyotype. (F) Summary of G-banded karyotype analyses of AG05397 fibroblast cells.

We assessed the copy number of chromosome 21, 14 days after Syn-mRNAs treatment by counting the number of signals (dots) in FISH, using a probe hybridizing to the centromeric region of chromosome 21 (see Materials and methods). In interphase nuclei, the presence of three fluorescent dots indicates three copies of chromosome 21 (Trisomy 21, DS), whereas the presence of two fluorescent dots indicates two copies (Fig. 3C). After scoring a large number of nuclei with either two or three dots in a blinded manner, the fraction of cells with two dots were calculated. The treatment of AG05397 DS cells with Syn-hZSCAN4 indeed increased the fraction of cells with two dots to 7%, whereas the treatment with a control Syn-GFP showed 1% of cells with two dots (Fig. 3D). These two dots in the control cells are most likely background noise in the FISH assays (see below for details).

The analyses thus far relied on counting only chromosome 21, which did not tell us whether other chromosomes were normal after exposure to ZSCAN4 biologics. Therefore, we examined the entire karyotype of these cells with high-resolution G-banded karyotype analyses in a blinded manner (Fig. 3E). Clinical cytologists reported that most cells carried trisomy 21, consistent with a diagnosis of DS, but found, oddly, that 2 nuclei out of 37 examined appeared normal, with no karyotype abnormality in one of three samples (Fig. 3F). It turned out that the cells with a normal karyotype were found only in Syn-hZSCAN4-treated DS cells, but not in non-treated control cells or Syn-GFP-treated control cells (Fig. 3F). These results were consistent with the results obtained by FISH, and also indicated that FISH may have background noise: 1% of Syn-GFP-treated cells showed two dots by FISH, but none were normal by G-banded karyotyping.

We repeated the analyses five times for AG05397 cells (47,XY,+21) and also extended the analyses to non-immortalized fibroblast cells derived from two individuals with DS in triplicate: AG06872 cells (47,XX,+21) and AG08942 cells (47,XY,+21) (see Materials and methods for details). The treatment with Syn-hZSCAN4 showed a statistically significant increase in cells with two dots (up to 14%) compared with control Syn-GFP (Fig. 4). The results indicate that the introduction of human ZSCAN4-mRNAs into cells can remove an extra copy of chromosome 21 without seeming to affecting other chromosomes.

**Figure 4.**
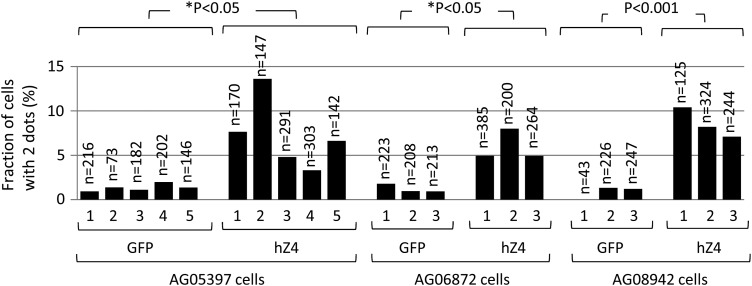
Analyses of three additional human trisomy 21 primary fibroblast cells with Syn-mRNAs. The fraction (%) of cells with two dots. The results of five biological replications for AG05397 cells and three biological replications for AG06872 and AG08942 cells are shown for each condition. For each sample, the numbers of nuclei examined to score two or three dots by FISH are shown above bars (*n*). GFP, cells treated with Syn-GFP; hZ4, cells treated with Syn-hZSCAN4. *P*, one-tailed *t*-test. P*, one-tailed Welch's *t*-test.

### 3.3. SeV vector expressing human ZSCAN4 increases euploid cells in DS fibroblast cells

As an independent method, we delivered ZSCAN4 to human cells with temperature-sensitive SeV vectors: SeV-TS15 (a permissive temperature of 35°C and non-permissive temperature of 37°C) and SeV-TS7 (a permissive temperature of 35°C, weakly functional temperature at 37°C, and non-permissive temperature at 39°C).^22^

First, we treated non-immortalized primary fibroblast cells isolated from individuals with DS (AG05397 and AG05024) with SeV-hZSCAN4-TS15. As controls, non-treated cells and cells treated with SeV-AG-TS15 were used. Immediately after plating cells (5 × 10^4^cells/well) in a 6-well plate, cells were treated with SeV-TS15 at 25 MOI (multiplicity of infection) (day 0), cultured at 35°C for 7 days, and then cultured continuously at 37°C for 2 weeks (Fig. 5A). On day 7, the production of proteins was monitored by fluorescence microscopy for SeV-AG-TS15 or by immunohistochemistry for SeV-hZSCAN4-TS15. In the third week of treatment (on day 21, 23, or 24), we assessed the fraction of cells with two dots (two copies of chromosome 21) by scoring more than 100 nuclei for each sample by FISH. Figure 5B shows a summary of results in three biological replications each: treating DS fibroblast cells with SeV-hZSCAN4-TS15 showed a statistically significant increase in cells with two dots over non-treated controls cells and cells treated with a control SeV-AG-TS15.

**Figure 5.**
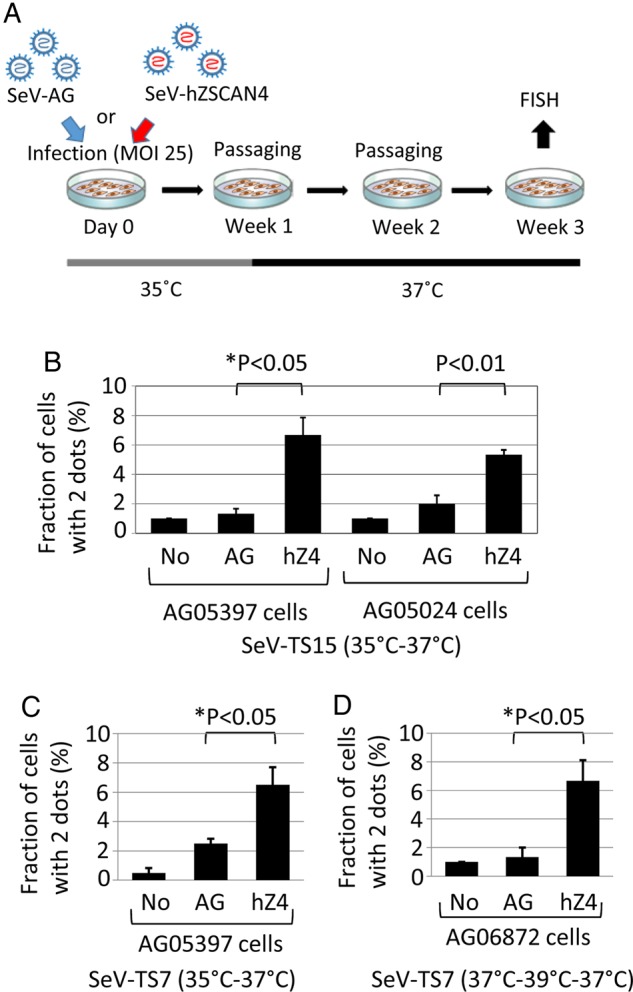
Analyses of human trisomy 21 primary fibroblast cells with SeV vectors. (A) Schematic overview of an experimental approach using SeV vectors. (B) The fraction (%) of AG05397 and AG05024 cells with two dots (mean ± SEM). The results of three biological replications are shown for each condition. For each sample, more than 200 nuclei were examined to score two or three dots by FISH. No, non-treated control; AG, cells treated with SeV-AG-TS15; hZ4, cells treated with SeV-hZSCAN4-TS15. Treatment by SeV-TS15 was carried out at 35°C. The cells were kept at 35°C for 7 days, followed by incubation at 37°C for 2 weeks. *P*, one-tailed *t*-test. P*, one-tailed Welch's *t*-test. (C) The fraction (%) of AG05397 cells with two dots (mean ± SEM). The results of three biological replications are shown for each condition. For each sample, more than 200 nuclei were examined to score two or three dots by FISH. No, non-treated control; AG, cells treated with a SeV-AG-TS7; hZ4, cells treated with a SeV-hZSCAN4-TS7. Treatment by SeV-TS7 was carried out at 35°C. The cells were kept at 35°C for 7 days, followed by incubation at 37°C for 2 weeks. P*, one-tailed Welch's *t*-test. (D) The fraction (%) of AG06872 cells with two dots (mean ± SEM). The results of three biological replications are shown for each condition. For each sample, more than 200 nuclei were examined to score two or three dots by FISH. No, non-treated control; AG, cells treated with SeV-AG-TS7; hZ4, cells treated with SeV-hZSCAN4-TS7. Treatment by SeV-TS7 was carried out at 37°C. The cells were kept at 37°C for 7 days, followed by incubation at 39°C for 3 days and then further incubation at 37°C for 11 days. P*, one-tailed Welch's *t*-test. This figure is available in black and white in print and in colour at *DNA Research* online.

Next, we tested SeV-hZSCAN4-TS7 in two different conditions: after the treatment of AG05397 DS cells, incubating cells at 35°C for 7 days, followed by the incubation at 37°C for 2 weeks (Fig. 5C); and after the treatment of AG06872 DS cells, incubating cells at 37°C for 7 days, followed by incubation at 39°C for 3 days, and further incubation at 37°C for 11 days (Fig. 5D). In both conditions, the treatment with SeV-hZSCAN4-TS7 showed a statistically significant increase in cells with two dots compared with non-treated control cells and cells treated with control SeV-AG-TS7.

To test the long-term effects of the treatments, we treated AG05397 (47,XY,+21) fibroblast cells with SeV-hZSCAN4-TS15 or SeV-AG-TS15 (control), cultured them at 35°C for 6 days, and subsequently continued culturing at 37°C (Fig. 6A). As a non-treated control, the same AG05397 cells were subjected to the same culture conditions except for the SeV-TS15 treatment. The analyses by immunohistochemistry on day 7 showed that ∼50–80% of cells were positive for AG or ZSCAN4, indicating the efficient delivery of ZSCAN4 protein to the cells (Fig. 6B). We sampled these cells and examined the number of chromosome 21 by FISH analyses every 1–2 weeks up to 8 weeks (Fig. 6A). As expected, after one treatment with SeV-hZSCAN4-TS15, the presence of cells with two dots had already increased in the second week and remained within the ranges between 8 and 24% until at least the eighth week (Fig. 6C). Strikingly, high-resolution G-banded karyotype analyses of cells sampled in the sixth week revealed that 41% (15 out of 37 nuclei examined) had a normal karyotype in a blinded experiment (Fig. 6D and E).

**Figure 6.**
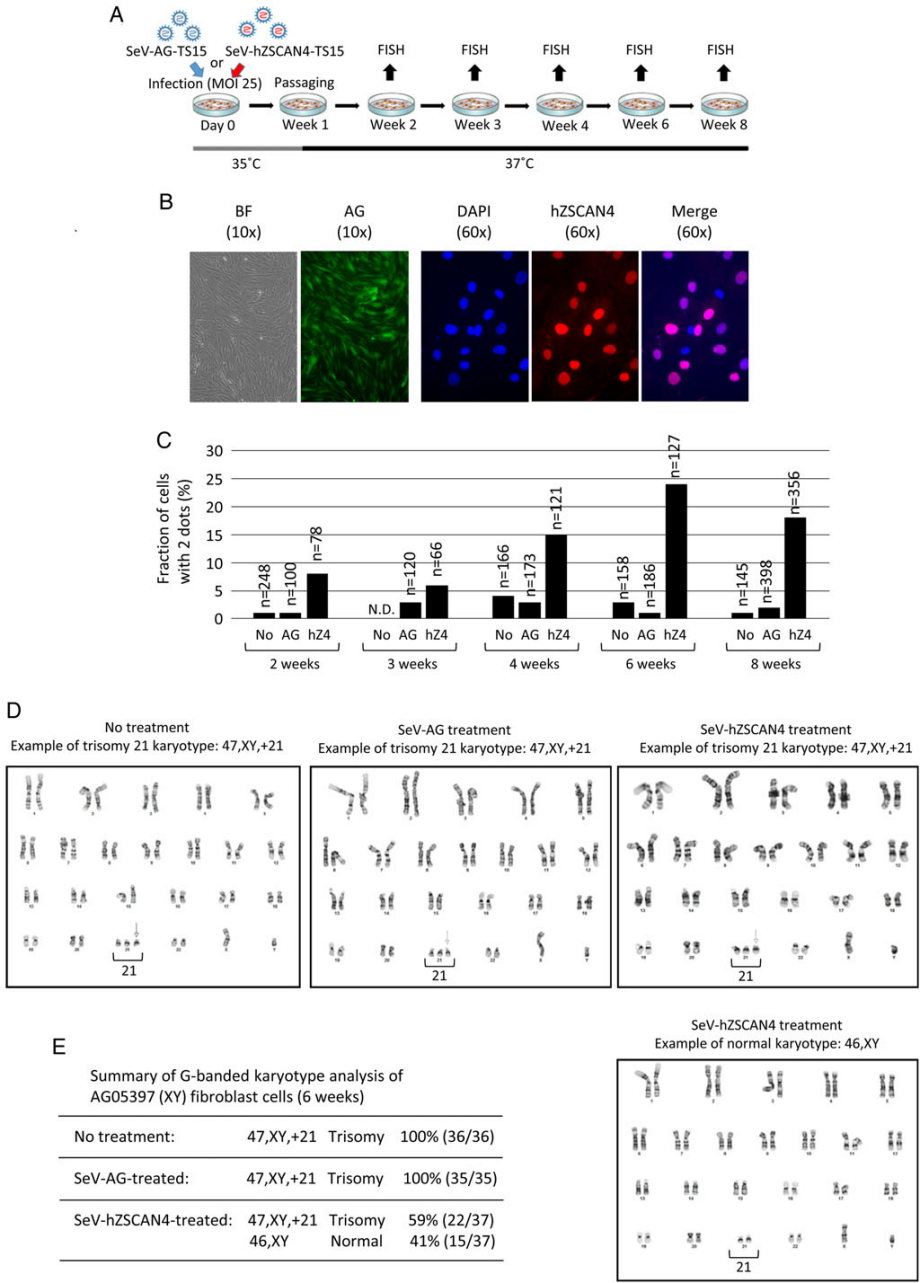
Analyses of the long-term effects of SeV vector treatment on human trisomy 21 primary fibroblasts. (A) Schematic overview of an experimental approach using SeV vectors for long-term cell cultures. The treatment by SeV-TS15 was carried out at 35°C. The cells were kept at 35°C for 6 days, followed by incubation at 37°C. Samples were taken every week and subjected to FISH analyses for counting the number of chromosome 21. (B) Assessment of AG expression by fluorescence microscopy and hZSCAN4 expression by immunohistochemistry using an antibody against human ZSCAN4. Day 7 after treating with SeV vector. BF, bright field. (C) The fraction (%) of cells with two dots. The numbers of nuclei examined to score two or three dots by FISH are shown above the bars (*n*). No, non-treated control; AG, cells treated with SeV-AG-TS15; hZ4, cells treated with SeV-hZSCAN4-TS15. (D) Examples of high-resolution G-banded karyotype analysis of AG05397 (trisomy 21) cells without treatment (No treatment), with a control SeV-AG treatment, or with SeV-hZSCAN4 treatment (upper, trisomy 21; lower, normal karyotype). (E) Summary of G-banded karyotype analysis of AG05397 fibroblast cells sampled in the sixth week.

### 3.4. Assessment of allelic balance by whole-exome sequencing

We further examined the integrity of the genome by carrying out 50-fold coverage whole-exome sequencing analyses on the cells sampled in the eighth week in a blinded test (Fig. 6A): non-treated control cells, SeV-AG-TS15-treated control cells, and SeV-hZSCAN4-TS15-treated cells. Sequences were compared with the reference human genome sequence (hg19) and sequence variants were identified by The Genome Analysis Toolkit (GATK).^25^ The quality of the obtained sequences and the detailed analysis pipeline are described in the Materials and methods section. The examination of variants of three samples for trisomic chromosome 21 as well as all other chromosomes revealed that these variants were biallelic, i.e. heterozygous or homozygous. The absence of the third alleles implied that the extra chromosome 21 was a duplicated copy of chromosome 21 of either parent. This also indicates that in all three samples, there was no contamination of cells derived from a person other than the donor of AG05397 (47,XY,+21) fibroblast cells.

To detect the changes of chromosome number in the cells, we used ‘AlleleBalance (AB)’—variant annotations provided by the GATK, which was calculated based on the read depth of a reference allele (REF) and an alternative allele (ALT).^25^ More specifically, to distinguish parental chromosomes, we used heterozygous AlleleBalance (ABHet), which was obtained by dividing the number of REF alleles by the total number of alleles at each variant locus throughout the human genome. In theory, the score of the ABHet should be close to 0.5 for heterozygous alleles. Figure 7 shows the histograms of trisomic chromosome 21 as well as comparably sized chromosomes 20 and 22. For both chromosomes 20 and 22, in all three samples, the distributions of the ABHet peaked around 0.5 as expected. In contrast, for chromosome 21, in non-treated control and SeV-AG-treated control samples, the distributions of the ABHet peaked between 0.3 and 0.4 and between 0.6 and 0.7. This implied that the distribution of ABHet was distorted by an extra chromosome 21. For example, if a REF allele is ‘A’ and an ALT allele is ‘G’, an expected ABHet score in trisomy 21 would be 0.33 (for the trisomic genotype AGG) or 0.66 (for the trisomic genotype AAG). Therefore, the two peaks were as expected. Interestingly, for the SeV-hZSCAN4-treated cells, the two peaks were reduced and the frequency of disomic alleles (0.5 peak) increased. This result was consistent with the presence of cells with both a normal disomic chromosome 21 and a trisomic chromosome 21 in the SeV-hZSCAN4-treated cells, although the exome sequencing of a single sample does not allow the quantitative estimation of the percentage of disomic cells in culture. Taken together, the exome sequencing analyses confirmed the removal of an entire chromosome 21 in some cells in culture by the ZSCAN4 biologics. In other words, the treatment with the ZSCAN4 biologics changed cells with homogeneously trisomy 21 into a mosaic of cells with trisomy 21 and cells with a normal karyotype.

**Figure 7.**
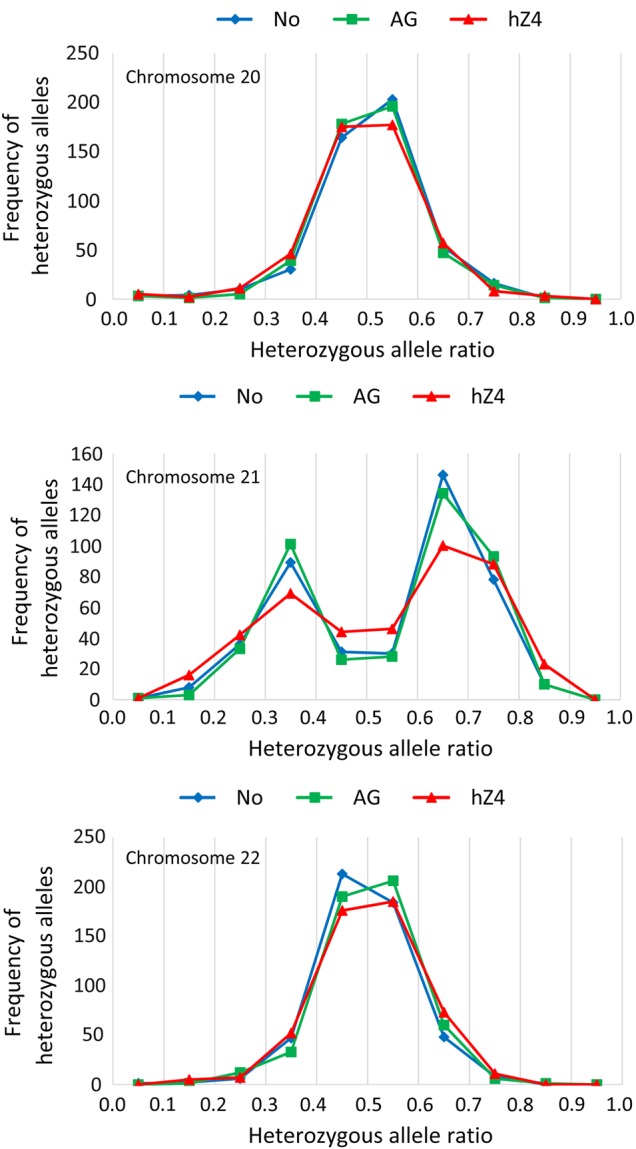
Whole-exome sequencing analysis. Analyses of DS fibroblast cells sampled from the eighth week (shown in Fig. 6A) by whole-exome sequencing. The frequency of heterozygous alleles for chromosome 20, 21, and 22 were plotted against the heterozygous allele ratio. No, non-treated control; AG, cells treated with SeV-AG-TS15; hZ4, cells treated with SeV-hZSCAN4-TS15.

Because whole-exome sequencing analyses do not provide telomere lengths—another important trait of the genome, we examined the telomere length on the same three samples after culturing for a more extended period. Telomere qPCR showed that SeV-hZSCAN4-treated cells indeed carried longer telomeres than control SeV-AG-treated cells or non-treated cells (Fig. 8A). The result was consistent with our earlier finding that ZSCAN4 can extend telomeres in mouse ES cells.^15^

**Figure 8.**
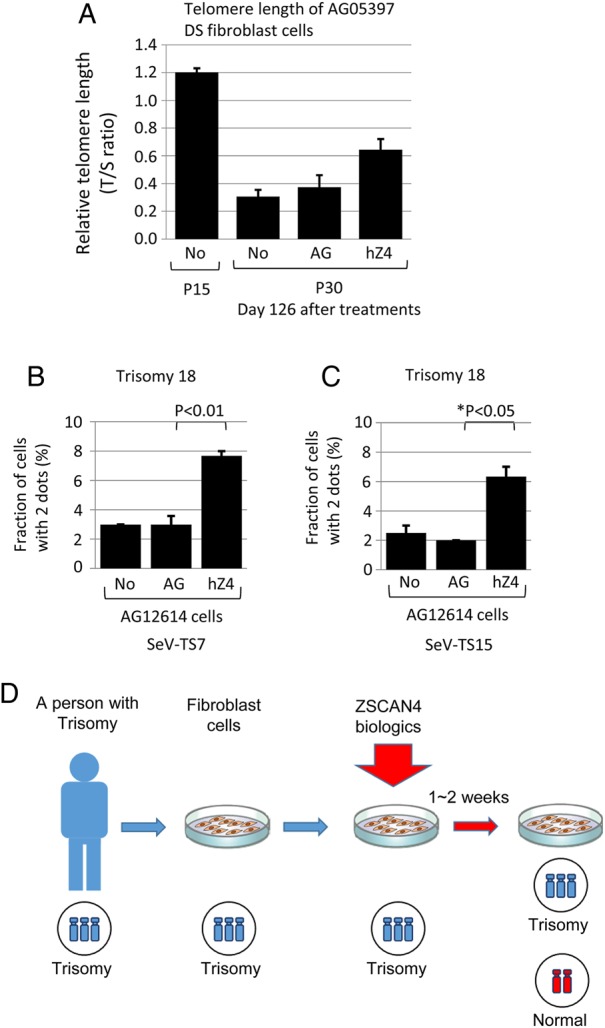
Analyses of trisomy 21 and trisomy 18 cells treated with SeV vectors. (A) Telomere lengths of the same cells analysed in Fig. 7. Telomere length was measured by qPCR and presented as the (T/S) ratio (telomere to single copy gene). Results of three technical replications are shown. Error bars (SEM). No (P15), non-treated control AG05397 cells at an early passage (passage 15); No (P30, day 126), non-treated control AG05397 cells at passage 30, day 126 in culture; AG (P30, day 126), cells treated with SeV-AG-TS15, cultured at 35°C for 6 days, followed by continuous culture at 37°C for 120 days. hZ4 (P30, day 126), cells treated with SeV-hZSCAN4-TS15, cultured at 35°C for 6 days, followed by continuous culture at 37°C for 120 days. Cells were passaged every week. (B) Treatment of trisomy 18 (Edwards syndrome). Non-immortalized fibroblast cells derived from an individual with trisomy 18 (AG12614) were treated with SeV-TS7. The cells were treated with SeV-TS7 at 37°C and cultured for 6 days, followed by incubation at 39°C for 3 days, and then, at 37°C for 15 days. The number of chromosome 18 was assessed by FISH using a chromosome 18-specific probe. Results of three biological replications are shown. The fraction (%) of cells with two dots are shown (mean ± SEM). For each sample, more than 200 nuclei were examined to score two or three dots by FISH. No, non-treated control; AG, cells treated with SeV-AG-TS7; hZ4, cells treated with SeV-hZSCAN4-TS7. *P*, one-tailed *t*-test. (C) Treatment of trisomy 18 (Edwards syndrome). Non-immortalized fibroblast cells derived from an individual with trisomy 18 (AG12614) were treated with SeV-TS15. The cells were treated with SeV-TS15 at 35°C and cultured for 6 days, followed by incubation at 37°C for 2 weeks. The number of chromosome 18 was assessed by FISH using a chromosome 18-specific probe. Results of three biological replications are shown. The fraction (%) of cells with two dots are shown (mean ± SEM). For each sample, more than 200 nuclei were examined to score two or three dots by FISH. No: non-treated control. AG, cells treated with SeV-AG-TS15; hZ4, cells treated with SeV-hZSCAN4-TS15. P*, one-tailed Welch's *t*-test. (D) Summary diagram showing the correction of trisomy.

### 3.5. ZSCAN4 biologics increase euploid cells in fibroblast cells derived from individuals with trisomy 18

To see whether ZSCAN4 can function comparably on other trisomic aneuploidies, we treated non-immortalized primary fibroblast cells (AG12614: 47,XX,+18) derived from an individual with trisomy 18—Edwards syndrome. First, we treated the cells in triplicate with SeV-hZSCAN4-TS7 and incubated them at 37°C for 6 days, followed by incubation at 39°C for 3 days and further incubation at 37°C for 12 days. FISH analyses using a chromosome 18-specific probe showed that SeV-mediated delivery of ZSCAN4 protein to the trisomy 18 cells increased the cells with two dots significantly over the non-treated control cells and cells treated with the control SeV-AG-TS7 (Fig. 8B). As an independent assessment, we treated the AG12614 trisomy 18 cells in triplicate with SeV-hZSCAN4-TS15 and incubated at 35°C for 6 days, followed by the incubation at 37°C for 15 days. Treatment with SeV-hZSCAN4-TS15 showed a statistically significant increase in cells with two dots over the non-treated control cells and cells treated with a control SeV-AG-TS15 (Fig. 8C). These results indicate that ZSCAN4 can increase euploid cells in cells with not only trisomy 21, but also trisomy 18.

## 4. Discussion

Human ZSCAN4 is surprisingly potent in increasing the number of euploid cells in mouse ES cells and non-immortalized human fibroblast cells carrying trisomy 21 or trisomy 18 (Fig. 8D). As far as we know, this is the first agent that can improve the karyotype of cells with chromosome abnormalities that have been considered to be irremediable.

### 4.1. ZSCAN4 biologics

We have produced and tested two kinds of ZSCAN4 biologics, i.e. synthetic mRNAs encoding mouse or human ZSCAN4 and Sendai virus vector expressing mouse or human ZSCAN4 for treatment *in vitro* and *in vivo*. Both kinds of biologics can transiently deliver ZSCAN4 to cells efficiently—one simply adds the biologics to the cells, leaving no footprint or exogenous DNA. These methods are particularly suitable to ZSCAN4, because it does not require constitutive or long-term expression to take effect. In fact, it functions normally, and in comparably short pulses, in early embryos and ES cells.

As we reported earlier, mouse Zscan4 genes comprise nine copies, one of which is the Zscan4c gene that has been used in the course of our study, whereas human ZSCAN4 is a single copy gene.^14^ Human ZSCAN4 is expressed in a subpopulation of tissue stem cells in the adult human pancreas^17^ and in human preimplantation embryos,^18^ but it has not been clear if the functions that we had seen for mouse Zscan4 could be applied to human genes and human cells. The results here show that in fact human ZSCAN4 is equally or more potent than mouse Zscan4 in human fibroblast cells and also in mouse ES cells. Thus, human ZSCAN4 biologics may be useful for experimentation in both human and mouse cells. Among the reagents, SeV-hZSCAN4-TS15 seems to be the most promising thus far, giving consistent effects and the highest efficiency. Optimum delivery vehicles, doses, timing, duration, and other conditions remain to be determined both *in vitro* culture and *in vivo* in the future.

### 4.2. Possible mechanisms

ZSCAN4 biologics rapidly improve karyotypes in mouse ES cells and human trisomic fibroblast cells, strongly suggesting that the ZSCAN4 protein corrects karyotype abnormalities directly and efficiently, presumably during the replication of the cells. Alternatively, one could imagine that ZSCAN4 might kill cells with an abnormal karyotype or suppress their proliferation, so that the proportion of cells with a normal karyotype would effectively increase. It could also be argued that spontaneously corrected euploid cells have a growth advantage over aneuploid cells and take over the culture over time. Such possibilities are, however, very unlikely. For mouse ES cells, we observed that karyotype improvement occurred within a few days without noticeable cell death. Although the forced expression of Zscan4 slows down the proliferation of mouse ES cells and mouse embryonic fibroblast cells, there has been no indication of aneuploidy-specific growth suppression.^33^ It has also been commonly observed that the proportion of aneuploid cells in mouse ES cell cultures increases over time.^16^ For human trisomic cells, we also observed the emergence of euploid cells without noticeable cell death in culture. Although recent reports noted that euploid cells grow faster than cells with trisomy 21,^10,11^ this does not seem to be the case in general according to detailed literature reviews.^3^ In this case, we should have seen a steady increase in the fraction of euploid cells in long-term culture after treating trisomy 21 cells with ZSCAN4 biologics; however, such a phenomenon was not observed (e.g. see Fig. 6C). It is also an unlikely explanation that ZSCAN4 specifically suppresses the proliferation of cells with trisomy, but not the proliferation of cells with a normal karyotype. For this explanation to be possible, all trisomic fibroblast cells that we have tested should have contained cells with a normal karyotype from the beginning (i.e. mosaicism) or should have gone through spontaneous correction of trisomy. However, these relatively rare events cannot account for all of the cases that we have studied here. With these explanations in mind, the most reasonable interpretation of the results presented in this report is that ZSCAN4 biologics directly correct karyotype abnormality, although other possibilities cannot be completely excluded at this point.

Given all of the cumulative data presented in this paper and previous work, what molecular mechanism explains the correction of karyotypes? Preliminarily, we speculate that ZSCAN4 biologics may correct aneuploidy based on mechanisms occurring naturally during organ development and maintenance. Indeed, anecdotal observations show that an abnormal karyotype is sometimes corrected during early preimplantation development,^34^ at the same time when mouse and human ZSCAN4 is highly and specifically expressed.^14,18^ A further hint about the mechanism comes from our earlier observations of Zscan4-mediated telomere elongation that increases genome stability in mouse ES cells.^15^ Interestingly, in the current study, we have seen comparable telomere elongation in human fibroblast cells by ZSCAN4 biologics, suggesting possible involvement of telomere regulation or steps in common with that process in the correction of karyotypes. Perhaps more importantly, the accompanying paper shows that transient and infrequent bursts of Zscan4 expression (named ‘Z4 events’) are accompanied by the rapid derepression of heterochromatin, particularly in the pericentromeric region, and the clustering of pericentromeric heterochromatin around the nucleolus.^35^ The accompanying paper also shows that at least some of these Zscan4 functions are mediated by forming complexes with both activating and repressing chromatin remodelling complexes,^35^ most likely through its SCAN domain for protein–protein interaction.^36^ In mouse ES cells, Zscan4 causes dramatic changes in the pericentromeric region—a fact is consistent with our findings reported here because the pericentromeric region is known to be critical for chromosome segregation and maintenance of a normal karyotype. However, it remains to be seen whether Z4 events can also occur in human fibroblast cells. The general implication is that during meiosis or mitosis, a ZSCAN4-mediated mechanism, possibly involving interactions with centromeres and/or telomeres, detects unpaired chromosomes and detaches them from the replication apparatus. Whatever the detailed mechanism, it now seems clear that ZSCAN4 is a material cause for the correction of aneuploidy: the mechanism operates independent of the vehicle of ZSCAN4 delivery to yield a dramatic increase in cells with normal karyotype.

### 4.3. Possible therapeutic applications of ZSCAN4 biologics

It is worth pointing out that irrespective of the mechanism of ZSCAN4 action, treating aneuploid cells with ZSCAN4 biologics increases the number of euploid cells. Its rapid action and high efficiency have allowed its direct application to non-immortalized primary human fibroblast cells. The potential efficacy of ZSCAN4 therapies is increased by the possibility that partial correction, as we have already seen, may have a significant effect. This is suggested by the milder DS phenotype conferred by mosaicism of trisomy 21 cells with a normal karyotype.^7^ In a similar way, direct ZSCAN4 treatment might provide alleviation of conditions.

ZSCAN4 biologics may also improve and maintain the quality of pluripotent stem cells in humans, mice, and other model organisms. One possible use is in mouse and human ES cells or iPS cells, which have been widely used to generate animal models for human diseases, or possible sources of regenerative tissue. Currently, their quality is often compromised during the long-term cell culture required for genetic engineering.

In summary, the results presented here suggest the possibility of a new therapy for genetic errors and chromosome abnormalities. As a naturally occurring protein that functions normally in very restricted timings and places during organ development and maintenance, it is conceivable that ZSCAN4 biologics may be developed into a ‘natural chromosome therapy’.

## Supplementary data

Supplementary data are available at www.dnaresearch.oxfordjournals.org.

## Funding

This work was in part supported by a grant from the Maryland Stem Cell Research Fund. Funding to pay the Open Access publication charges for this article was provided by the Maryland Stem Cell Research Fund and Elixirgen, LLC.

## Supplementary Material

Supplementary Data
