# Supplementary material for: Correction of Down syndrome and Edwards syndrome aneuploidies in human cell cultures
Source: DNA Res. 2015 Aug 31;22(5):331–42. doi: 10.1093/dnares/dsv016 (PMC4596399; doi:10.1093/dnares/dsv016)
Supplement: Supplementary Data [file supp_22_5_331__index.html]

Correction of Down syndrome and Edwards syndrome aneuploidies in human cell cultures — Supplementary Data 

# Correction of Down syndrome and Edwards syndrome aneuploidies in human cell cultures

## Supplementary Data

Supplementary Data

- Supplementary Data - Pdf file
