# Supplementary material for: Correction of Down syndrome and Edwards syndrome aneuploidies in human cell cultures
Source: DNA Res. 2015 Aug 31;22(5):331–42. doi: 10.1093/dnares/dsv016 (PMC4596399; doi:10.1093/dnares/dsv016)
Supplement: Supplementary Data [file supp_dsv016_dsv016supp.pdf]

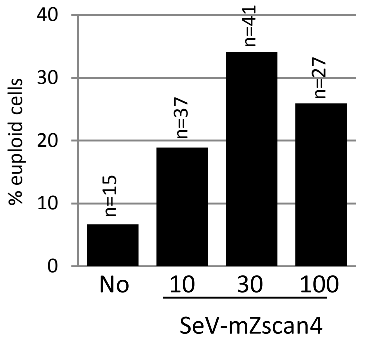

Supplementary Fig. S1. Optimization of SeV vector doses for mouse ES cells. Mouse ES cells (passage 33) were treated with SeV-mZscan4 with the multiplicity of infection (MOI) of 10, 30 and 100, respectively. Four days after the treatment, ES cells were karyotyped. The results were consistent with the previous report: the MOI of 30 was selected for human fibroblast cells (Ban et al., 2011). Based on these results, we selected the MOI of 30 (or 25) as our standard MOI for the SeV vector treatment.

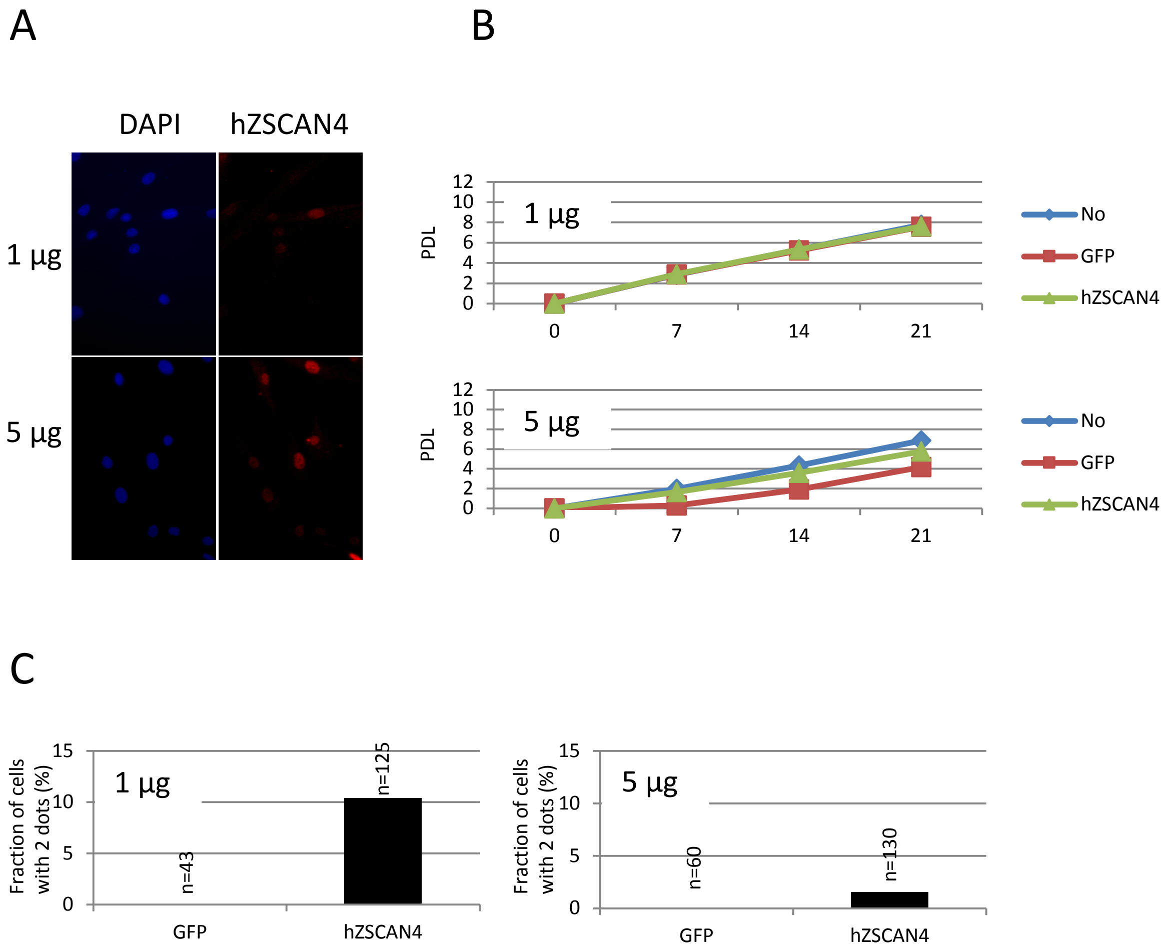

Supplementary Fig. S2. Optimization of Syn-mRNA doses. (A) AG08942 DS human fibroblast cells ($5x10^4$ cells/well) were plated in a 6-well plate and transfected with 1 µg (upper) and 5 µg (lower) of Syn-GFP and Syn–hZSCAN4, respectively. Next day, cells were stained with hZSCAN4 antibody. Nuclei was visualized by DAPI. Cells transfected with 5 µg mRNA showed stronger signals of hZSCAN4 than those transfected with 1 µg mRNA. (B) Proliferation profiles of cells. Cells were passaged every week and cultured for 3 weeks. PDL (population doubling level) was calculated in every passaging. Lower PDL of cells transfected with 5 µg of Syn-mRNAs compared to cells transfected with 1 µg of Syn-mRNAs was due to the death of many cells on day 1. (C) FISH analyses. On day 14, cells were harvested for the FISH analyses. The treatment of cells with 1 µg Syn-hZSCAN4 increased the fraction of cells with 2-dots to 10% (left), whereas the treatment of cells with 5 µg of Syn-hZSCAN4 showed only 1% of cells with 2 dots (right). We selected 1 µg of Syn-hZSCAN4 for all the subsequent experiments. This was the standard amount of Syn-mRNAs used in the previous publication (Warrant et al., 2010).

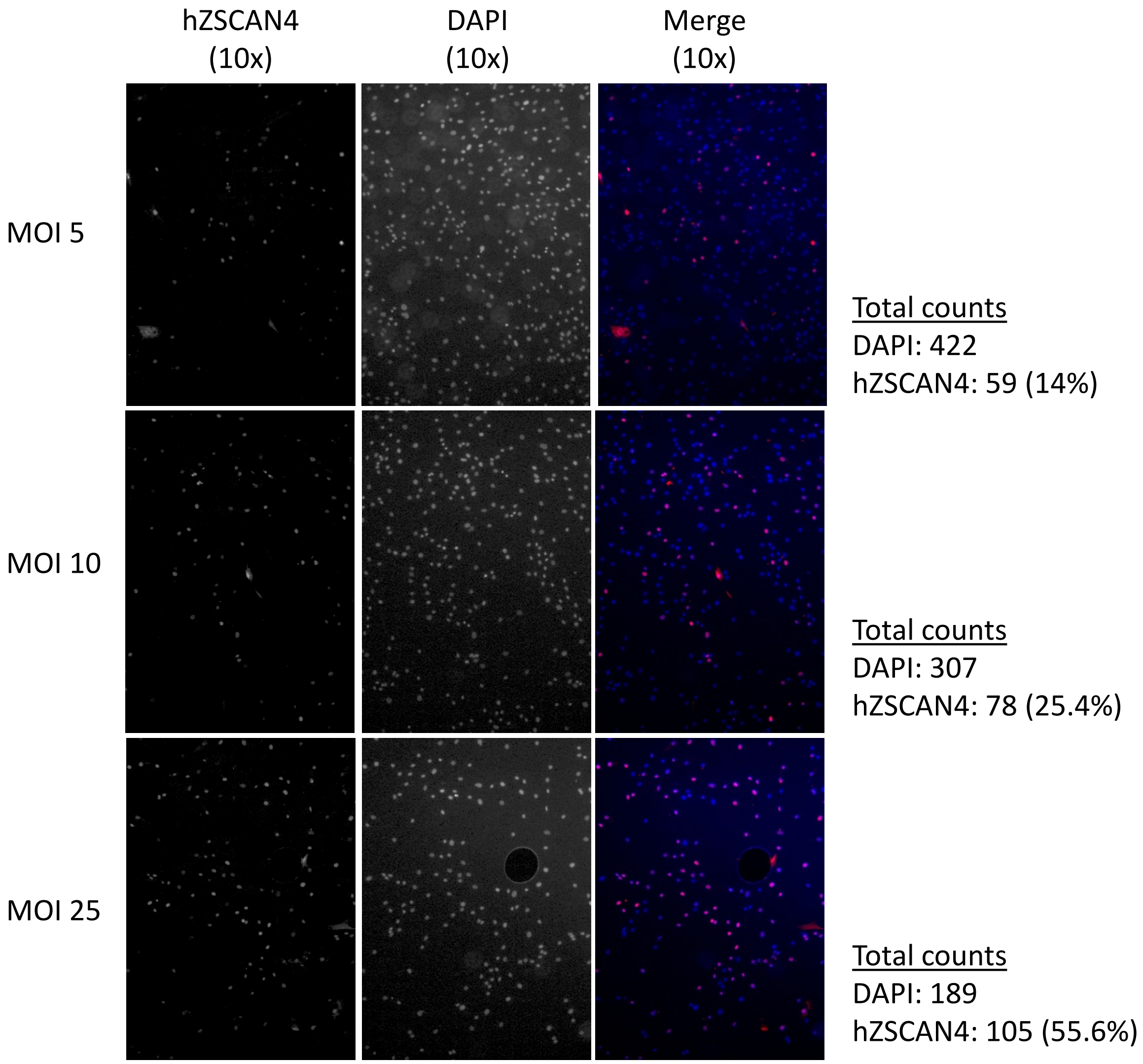

Supplementary Fig. S3. Optimization of SeV vector doses for human fibroblast cells. AG05397 DS fibroblast cells (passage 21) were treated with SeV-hZSCAN4-TS15 at the MOI of 5, 10, and 25, respectively. Expression of hZSCAN4 was monitored 7 days after the treatment by the immunohistochemistry using an antibody against human ZSCAN4. The results were consistent with the previous report: the MOI of 30 was selected for human fibroblast cells (Ban et al., 2011). Based on these results, we selected MOI=25 as the standard MOI for all subsequent experiments.
